# Recent adverse trends in semen quality and testis cancer incidence among Finnish men

**DOI:** 10.1111/j.1365-2605.2010.01133.x

**Published:** 2011-08

**Authors:** N Jørgensen, M Vierula, R Jacobsen, E Pukkala, A Perheentupa, H E Virtanen, N E Skakkebæk, J Toppari

**Affiliations:** * University Department of Growth and ReproductionRigshospitalet, Copenhagen, Denmark; † Department of Physiology, University of TurkuTurku, Finland; ‡ Institute of Public Health, Epidemiology, University of Southern DenmarkOdense, Denmark; § Finnish Cancer Registry, Institute for Statistical and Epidemiological Cancer ResearchHelsinki, Finland; ¶ Department of Obstetrics and Gynaecology, University of TurkuTurku, Finland; ** Department of Paediatrics, University of TurkuTurku, Finland

**Keywords:** birth cohort effects, Finland, general population, semen quality, sperm, time trends

## Abstract

Impaired semen quality and testicular cancer may be linked through a testicular dysgenesis syndrome of foetal origin. The incidence of testis cancer has been shown to increase among Finnish men, whereas there is no recent publication describing temporal trends in semen quality. Therefore, we carried out a prospective semen quality study and a registry study of testis cancer incidence among Finnish men to explore recent trends. A total of 858 men were investigated in the semen quality study during 1998–2006. Median sperm concentrations were 67 (95% CI 57–80) million/mL, 60 (51–71) and 48 (39–60) for birth cohorts 1979–81, 1982–83 and 1987; total sperm counts 227 (189–272) million, 202 (170–240) and 165 (132–207); total number of morphologically normal spermatozoa 18 (14–23) million, 15 (12–19) and 11 (8–15). Men aged 10–59 years at the time of diagnosis with testicular cancer during 1954–2008 were included in the registry study, which confirmed the increasing incidence of testicular cancer in recent cohorts. These simultaneous and rapidly occurring adverse trends suggest that the underlying causes are environmental and, as such, preventable. Our findings necessitate not only further surveillance of male reproductive health but also research to detect and remove the underlying factors.

## Introduction

Testis cancer rates have increased in western countries although with significant variations between countries (Chia *et al.*, 2010; Engholm *et al.*, 2010). Thus, for several decades, the incidence rates among Finnish men were lower than for men from most other countries, and at the same time, the Finnish men were found to have higher sperm counts than men from other Nordic countries (Vierula *et al.*, 1996; Jørgensen *et al.*, 2002). However, recent publications have indicated a change towards accelerated testis cancer rates among Finns (Richiardi *et al.*, 2004).

Testis cancer and other male reproductive problems including impaired semen quality have been suggested to be linked through a testicular dysgenesis syndrome of foetal origin due to impact of environmental factors (Skakkebæk *et al.*, 2001). Thus, such reproductive health problems may be related to year of birth, although not manifested until adulthood.

A question is whether the reported better reproductive health of Finnish men could be due to different environmental exposures or caused by genetic differences. We hypothesized that the better situation in Finland could be due to the fact that Finland ‘lagged behind’ in exposures to modern industrial pollution. Therefore, we carried out semen studies on cohorts of young Finnish men, and a registry study of testis cancer to explore recent temporal trends.

## Materials and methods

### Semen quality study

The inclusion criteria and investigation methods of the young Finnish men were similar to those applied in the previously published studies (Jørgensen *et al.*, 2002).

#### Study populations

Finnish young men are required to attend a medical examination when they are 18 or 19 years. Such groups can be considered representative of the general population, and we invited all such men to our study utilizing the same drafting lists irrespective of whether the men were fit or unfit for military service. Hence, all men were sent a letter inviting them to join the present study, with replies returned either over the telephone or by post. Further inclusion criteria were that the men lived in the Turku area in Finland and that they and their mothers were born in Finland. For men agreeing to participate, a subsequent appointment for examination at the Turku University Andrology Unit in the Institute of Biomedicine was made. Each participant completed a questionnaire (including information on age, previous or current diseases and some life-style factors), underwent a physical examination, provided a semen sample and received financial compensation (40–50 Euro). The study period was 1998–2006. Altogether, 858 men were investigated. Results for 324 of these have previously been reported (Jørgensen *et al.*, 2002), but were also included here to describe temporal changes. The participation rate was 13.4%.

A description of the study population based on questionnaire information and results from the physical examination is given in Table 1. The three types of information in Table 1‘Been diagnosed as having’, ‘Been treated for’ and ‘Has’ are based on questions phrased as ‘Has a doctor ever diagnosed you as having…’, ‘Have you ever been treated for…’ and ‘Have you ever…’respectively. During the preceding 3 months to participation in the study, 17.6% had used medication, which mainly were antibiotics, painkillers, asthma or allergy medicine. Prior to participation in the study, 1.2% of the men had been diagnosed with a varicocoele and 0.8% had been operated. Few men had been treated for a disease of penis, urethra or scrotum (Table 1). All specified treatments were due to phimosis. None of the men answered positively to have been treated for hypospadias. During the physical examination, the penises of all men were classified as normal, and without signs of previous treatments of hypospadias, and none was diagnosed with hypospadias.

**Table 1:** Physical appearance and self-reported information of young men from the Turku area in Finland

	Investigation period								
	
	1998–2006, total (*N*= 858)		1998–99 (*N*= 338)		2001–03 (*N*= 382)		2006 (*N*= 138)		
					
	Mean (SD)	Median (5–95)	Mean (SD)	Median (5–95)	Mean (SD)	Median (5–95)	Mean (SD)	Median (5–95)	*p*-value
Physical appearance:									
Age (years)^a^	19.0 (0.4)	19.0 (18.3–19.6)	18.8 (0.5)	18.8 (18.2–19.6)	19.0 (0.4)	19.1 (18.4–19.7)	19.1 (0.1)	19.1 (18.9–19.4)	<0.005^A^
Height (m)	1.79 (0.1)	1.80 (1.70–1.90)	1.80 (0.1)	1.80 (1.70–1.91)	1.79 (0.1)	1.80 (1.70–1.90)	1.79 (0.1)	1.80 (1.70–1.90)	0.6 A
Weight (kg)	74 (11)	72 (59–95)	73 (11)	72 (57–95)	74 (11)	73 (58–95)	74 (11)	73 (60–93)	0.4 A
BMI (kg/m^2^)	22.8 (3.0)	22.5 (18.7–28.4)	22.6 (2.8)	22.2 (18.6–28.2)	23.0 (3.1)	22.6 (18.7–28.1)	23.2 (3.0)	22.7 (19.1–29.0)	0.08 A
Testis size (mL)^b^	22 (4)	21 (15–28)	22 (4)	22 (15–29)	21 (4)	21 (15–27)	21 (4)	21 (15–25)	0.07 A
Life-style:									
Cigarettes daily, all men	4.2 (6.4)	0.0 (0.0–19.5)	4.1 (6.3)	0.0 (0.0–20.0)	4.5 (6.6)	0.0 (0.0–19.7)	3.8 (6.0)	0.0 (0.0–15.8)	0.5 A
Cigarettes daily, smokers only	10.7 (5.8)	10.0 (2.0–20.0)	10.5 (5.9)	10.0 (1.0–20.0)	10.8 (6.0)	10.0 (1.5–20.0)	11.1 (5.0)	10.0 (2.4–20.0)	0.7 A
Alcohol consumption (units)^c^	9.7 (11.1)	7.0 (0.0–30.0)	9.1 (10.1)	7.0 (0.0–30.0)	10.0 (12.2)	7.0 (0.0–30.5)	10.4 (10.0)	9.0 (0.0–28.15)	0.5 A

	%		%		%		%		
Smokers	41.7		42.8		43.4		34.3		0.2^B^
Mother smoked in pregnancy	10.2		11.4		10		7.8		0.5 B
Been diagnosed as having:									
Varicocoele	1.2		2.1		0.5		0.8		0.1 B
Epididymitis	0.5		0.6		0.3		0.8		0.7 B
Sexually transmitted disease^d^	1.5		1.6		1.4		1.5		0.97 B
Cystitis	2.5		2.3		2.5		3.1		0.9 B
Diabetes	0.3		0.0		0.0		0.8		0.08 B
Thyroid disease	0.4		0.3		0.6		0.0		0.6 B
Been treated for:									
Varicocoele	0.8		1.5		0.5		0.0		0.2 B
Testicular torsion	0.8		0.9		0.8		0.7		0.98 B
Testicular cancer	0.0		0.0		0.0		0.0		–
Other diseases of penis, urethra or scrotum^e^	2.5		2.4		2.4		3.0		0.9 B
Inguinal hernia	5.1		6.8		3.9		4.4		0.2 B
Has:									
Had cryptorchidism^f^	7.8		10.1		6.1		6.7		0.1 B
Experienced fertility problems^g^	0.8		0.9		0.8		0.0		0.9 B
Caused a pregnancy^h^	4.0		2.4		5.8		3.0		0.06 B
taken any medication during past 3 months^i^	17.6		15.7		18.1		21.0		0.4 B
Subgroup of men not affected by any of above three main conditions:	63.2		63.0		65.4		57.2		0.2^B^

SD: Standard deviation; 5–95: 5–95 percentiles.

Data on physical appearance were obtained during the physical examination of the study subjects, whereas the remaining were obtained from the questionnaire answered by the men.

The three groups ‘Been diagnosed as having’, ‘Been treated for’ and ‘Has’ are based on questions phrased as ‘Has a doctor ever diagnosed you as having…’, ‘Have you ever been treated for…’ and ‘Have you ever…’ respectively.

The ‘Subgroup of men not affected by any of above three main conditions’ are men who were not included in the above-mentioned three groups.

a Calculated as difference between day of attendance in study and self-reported day of birth.

b Mean of left and right testes size assessed by palpation. Information of testis size was missing for 3, 9 and 3 men from the 1st, 2nd and 3rd investigation period, respectively.

c Sum of intake of beer, wine and strong alcohol recent week prior to participation in study.

d Chlamydia or gonorrhoea.

e If answer was yes, then the men were asked to specify the type of treatment, which were all treatment for phimosis. None reported being treated for hypospadias.

f Ever had cryptorchidism, i.e. not born with both testicles in scrotum (irrespective of spontaneous descend, treatment or still cryptorchid).

g Ever had regular intercourse without use of contraception for at least one year, without partner became pregnant.

h Ever caused a pregnancy.

i Taken any medication recent 3 months prior to participation in study.

A Kruskal–Wallis.

B Chi-squared test.

*p*-value: For comparison of results among the three study periods. The difference in age was caused by younger age of the men investigated 1998–99.

A subgroup of men who had not taken any medicines and were without any previous or current andrological diseases, infertility problems or known fertility were defined, and they constituted 63.2% of the entire study group (Table 1).

#### Semen samples

Semen samples were obtained by masturbation in the privacy of a room adjacent to the laboratory. Ejaculation abstinence of at least 48 h was recommended and the abstinence periods as reported by the men were recorded. Semen volume was estimated by weighing. For sperm motility assessment, duplicates of 10 μL of well-mixed semen were placed on a glass slide, examined under a microscope at ×400 magnification and spermatozoa were classified as motile or immotile. The average of the two countings was used. For the assessment of the sperm concentration, the samples were diluted in a solution of 0.6 mol/L NaHCO_3_ and 0.4% (v/v) formaldehyde in distilled water, and subsequently assessed using an improved Neubauer haemocytometer. Finally, smears were prepared, Papanicolaou stained and assessed according to strict criteria (Menkveld *et al.*, 1990). The same technician assessed semen volume, sperm motility and concentration during all the study years. Assessments of morphology were performed by one investigator (MV) at the end of the study period. Thus, also samples that previously had been assessed by another investigator (Jørgensen *et al.*, 2002) were reassessed.

A continuous external quality control programme for assessment of sperm concentration (Jørgensen *et al.*, 2002; Punab *et al.*, 2002; Paasch *et al.*, 2008) was performed. In the period 1998–2006, five quality control samples were sent to the participating centres including the Turku laboratory 8–10 times yearly, and assessed as described above. Thus, in the study period, more than 400 samples were assessed in the QC programme. The results showed that the Turku laboratory constantly assessed 11% higher than the Copenhagen reference laboratory (95% confidence interval 2–22%, *p*= 0.02), consistent with the result previously reported (Jørgensen *et al.*, 2002). Thus, for description of temporal changes, no adjustments according to these quality control results were needed because of the constant level.

#### Physical examination

Testis sizes were measured using a Prader orchidometer, presence of varicocoele or other scrotal abnormalities and the Tanner stage of pubic hair was evaluated. Body height and weight were measured, and body mass index (BMI) was calculated. One physician undertook 82% and another 15% of the examinations, and the deputies, the remaining 3%.

#### Participants vs. non-participants

Information about the total number of eligible men and zip-code of their current place of living was obtained.

#### Statistical analysis – semen study

Standard statistics (mean, median, standard deviation (SD), 5–95 percentiles and frequencies) were used for basic descriptions. The study subjects were described as three groups depending on the investigation periods: first group 1998–99 (examined February 1998 to January 2000), second group 2001–2003 (examined December 2000 to April 2003) and third group 2006 (examined April 2006 to January 2007). Between-group differences for continuous variables were tested using the non-parametric Kruskal–Wallis test. Between-group differences for categorical variables were tested using the Pearson chi-squared test.

The main outcome variables were the semen variables. Both temporal trends according to the investigation periods and according to birth cohorts (described as three groups born 1979–81, 1982–83 and 1987 respectively) were tested by multiple linear regressions adjusted for confounders. Semen volume, sperm concentration and total sperm counts were normalized by cubic root transformation before analysis to correct for skewed distribution of residuals. The percentages of motile spermatozoa were logit-transformed. Percentages of morphologically normal spermatozoa entered the model untransformed. Ejaculation abstinence up to 96 h had an increasing effect on semen volume, sperm concentrations and total sperm counts (all *p*< 0.05), and was entered as a covariate in the regression analyses of these variables, whereas it had no effect on either morphology or motility. There was a tendency for increasing frequency of morphologically normal spermatozoa with increasing age (beta 0.59% per year), although non-significant (*p*= 0.17). However, in the final models, all semen variables were adjusted for age. Season of year was evaluated as a possible confounder for all the semen variables. For sperm motility, duration from ejaculation to assessment was additionally evaluated as a confounder. Both were non-significant and therefore not included.

Natural logarithmic transformation gave models in which period differences and effects of covariates are more easily interpretable. This alternative model approximates closely the model obtained by cubic root transformation and is used when reporting adjusted semen volumes, sperm concentrations, total sperm counts and total number of morphologically normal spermatozoa, adjusted to represent a 19-year-old man having an ejaculation abstinence period of 96 h. The logit-transformed motility data and untransformed morphology percentages were used to give adjusted levels for a 19-year-old man for these variables.

*p*< 0.05 was considered statistically significant. Analyses were performed using PASW GradPack 18.0 (SPSS Inc., Chicago, IL, USA).

### Registry study of testicular cancer

Data on incident testicular cancer cases were gathered from the Finnish Cancer Registries. In the period 1954–2008, 5974 men were diagnosed with testicular cancer. Among these, 484 were younger than 10 years-old and 264 were aged more than 60 years. The remaining (*N*= 5226) were included for calculation of age-specific and age-adjusted incidence rates (per 100000) and described by cumulated incidence rates for men from the whole of Finland, and for the Turku region (the part of the South-West part where the semen quality study was undertaken). Among the 5226 men, 1820 were aged 10–19 years, 1804 20–29 years, 886 30–39 years, 478 40–49 years and 238 50–59 years at the time of diagnosis of testicular cancer. From the Turku area, of the 800 men aged 10–59 years who were diagnosed with testicular cancer in 1954–2008, 282 were aged 10–19 years, 270 20–29 years, 128 30–39 years, 92 40–49 years and 28 50–59 years. Approximate birth cohorts born in 1930–38 were used for the analyses of the age-specific trends. Birth cohorts were computed for men aged 10–59 years.

## Results

### Study population

When testing for differences in physical appearance and self-reported information, only age differed significantly among the three study groups (Table 1). The following pair-wise testing showed this to be caused by the first study group that was marginally younger than the latter two (18.8 vs. 19.1 years). Otherwise, there were no significant differences between the groups in physical appearance, life-style or medical history.

### Semen quality study

#### Semen quality

Semen results according to investigation periods are shown in Table 2. ‘Observed’ values are based on raw data, and ‘Adjusted’ are the confounder-adjusted estimates obtained from regression analyses. The median ejaculation abstinence was 65 h (64, 65 and 67 for the groups 1998–99, 2001–03 and 2006 respectively). Semen volume did not differ among the three investigation periods, whereas sperm concentration, total sperm counts, percentage of morphologically normal spermatozoa and total number of morphologically normal spermatozoa all decreased. The percentages of motile spermatozoa varied without any specific trend (medians 65–74%). The same calculations as presented in Table 2 were made on the subgroup of men not affected by any of the conditions defined in Table 1, and for this subgroup, a temporal trend similar to that for the entire study population was seen (Table 3).

**Table 2:** Semen quality of young men from the Turku area in Finland

	Observed		Adjusted	*p*-values comparing investigation periods^A^		
			
	Mean (SD)	Median (5–95)	Median (95% CI)	All periods	2006 vs. 2001–03	2006 vs. 1998–00
Semen volume (mL):						
Investigation period 1998–99	3.3 (1.4)	3.0 (1.3–5.6)	3.4 (3.1–3.7)	0.99	0.97	0.97
Investigation period 2001–03	3.3 (1.5)	3.1 (1.2–6.1)	3.4 (3.1–3.6)			
Investigation period 2006	3.4 (1.5)	3.3 (1.3–6.3)	3.3 (3.0–3.7)			
Sperm concentration (million/mL):						
Investigation period 1998–99	71 (59)	60 (3–185)	67 (57–80)	0.04	0.02	0.02
Investigation period 2001–03	68 (65)	54 (5–167)	60 (51–71)			
Investigation period 2006	58 (50)	50 (1–141)	48 (39–60)			
Total sperm count (million):						
Investigation period 1998–99	219 (175)	193 (12–519)	228 (190–274)	0.07	0.04	0.03
Investigation period 2001–03	212 (201)	174 (17–522)	201 (169–238)			
Investigation period 2006	183 (140)	156 (2–450)	165 (132–207)			
Normal morphology (%):						
Investigation period 1998–99	9.7 (5.3)	9.0 (2.0–19.2)	9.8 (9.2–10.4)	0.05	0.3	0.03
Investigation period 2001–03	9.1 (5.3)	8.5 (1.7–18.5)	9.1 (8.5–9.7)			
Investigation period 2006	8.7 (4.9)	8.5 (1.5–18.0)	8.6 (7.6–9.5)			
Total normal spermatozoa (million):						
Investigation period 1998–99	24 (25)	17 (0.6–70)	18 (14–23)	0.02	0.09	0.006
Investigation period 2001–03	22 (30)	14 (0.5–62)	15 (12–19)			
Investigation period 2006	18 (18)	10 (0.4–55)	11 (8–15)			
Motile (%):						
Investigation period 1998–99	63 (15)	66 (37–80)	65 (63–66)	<0.0005	0.7	<0.0005
Investigation period 2001–03	72 (13)	75 (45–86)	74 (72–75)			
Investigation period 2006	70 (19)	74 (31–89)	73 (71–74)			

Observed: Results based on raw data.

SD: Standard deviation; 5–95: 5–95 percentiles.

Adjusted median and 95%CI (confidence interval) calculated by linear regression analysis.

Sperm concentration, total sperm and semen volume adjusted to a period of ejaculation of 95 h for a 19-year-old man. Motility and morphology were also adjusted to represent a 19-year-old man. See text for further details.

A Linear regression analyses, adjusted for co-variates as described above.

**Table 3:** Semen quality of the entire study group young men (‘all men’) from the Turku area in Finland, and a subgroup of these without previous or current conditions that potentially could affect the semen quality

	Adjusted median (95% CI) for investigation periods		
	
	1998–99	2001–03	2006
Semen volume (mL):			
All men	3.4 (3.1–3.7)	3.4 (3.1–3.6)	3.3 (3.0–3.7)
Subgroup	3.4 (3.0–3.7)	3.4 (3.1–3.8)	3.4 (3.0–3.9)
Sperm concentration (million/mL):			
All men	67 (57–80)	60 (51–71)	48 (39–60)
Subgroup	65 (52–819	58 (47–72)	48 (36–64)
Total sperm count (million):			
All men	228 (190–274)	201 (169–238)	165 (132–207)
Subgroup	218 (173–275)	200 (160–249)	172 (128–231)
Normal morphology (%):			
All men	9.8 (9.2–10.4)	9.1 (8.5–9.7)	8.6 (7.6–9.5)
Subgroup	9.8 (9.0–10.6)	9.0 (8.3–9.7)	8.9 (7.6–10.1)
Total normal spermatozoa (million):			
All men	18 (14–23)	15 (12–19)	11 (8–15)
Subgroup	18 (14–25)	15 (11–20)	13 (9–20)
Motile (%):			
All men	65 (63–66)	74 (72–75)	73 (71–74)
Subgroup	65 (63–67)	74 (72–76)	73 (71–76)

For definition of the subgroup, see Table 1.

Adjusted median and 95% CI (confidence interval) calculated by linear regression analysis.

Sperm concentration, total sperm and semen volume adjusted to a period of ejaculation of 95 h for a 19-year-old man. Motility and morphology were also adjusted to represent a 19-year-old man. See text for further details.

For the entire study group, 13 and 36% had sperm concentrations below 15 and 40 million/mL, and 55 and 86% had less than 9 and 12% morphologically normal spermatozoa respectively.

**Figure 1:**
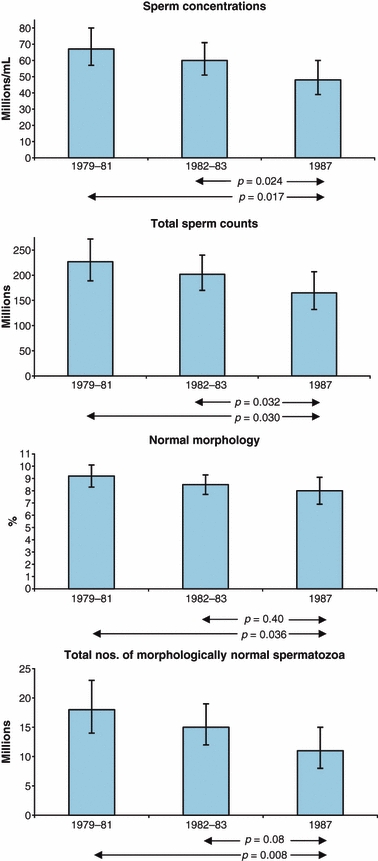
Sperm concentrations, total sperm counts and morphology according to year of birth of men representative of the general young Finnish population. The figures represent medians adjusted for confounders and 95% confidence intervals. The most recently born men had significantly lower levels than the earliest born men.

**Figure 2:**
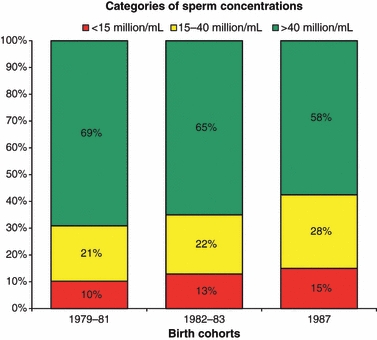
Frequencies of young Finnish men having sperm concentrations below 15, 15–40 and above 40 million/mL in three birth cohorts. Note the trends for more men having sperm concentrations below 40 million/mL; 31, 35 and 43% for the birth cohorts 1979–81, 1982–83 and 1987 respectively.

The birth years for the participants ranged from 1979 to 1987; 39, 45 and 16% were born in 1979–81, 1982–83 and 1987, respectively. Figure 1 illustrates the adjusted levels of sperm concentrations, total sperm counts and morphology according to the birth year categories in contrast to the Tables where the results were stratified according to the investigation period. The most recently born men had lower levels of semen variables than the earliest born. Based on men who had ejaculation abstinence of at least 48 h, Fig. 2 illustrates that the frequencies of men having sperm concentrations below 15 million/mL and in the range 15–40 million/mL increased in the recent birth cohorts.

Men who reported that their mothers had been smoking during pregnancy (*N*= 10.2%) did not significantly differ for their semen variables from non-exposed men (adjusted median semen volume 3.1 vs. 3.4 mL, *p*= 0.3; total sperm count 207 vs. 209 million, *p*= 0.6; percent motile spermatozoa 67 vs. 69%, *p*= 0.3; sperm concentration 64 vs. 62 million/mL, *p*= 0.8; percentage of morphologically normal spermatozoa 8.6 vs. 8.6%, *p*= 0.9). Similarly, the men's own smoking or drinking habits did not affect the semen variables.

The participants were categorized into two based on their current place of living, Turku City and surrounding areas (neighbouring municipalities Kaarina and Raisio). Men from ‘surrounding areas’ had a frequency of motile spermatozoa at 72%, *p*= 0.001, in comparison with men living in Turku City (comparison level 100%). No effect of place of living could be detected for other semen variables, *p*= 0.4–0.9.

#### Physical examination

The median (5–95 percentiles) sizes of the left and right testes were 20 mL (14–27 mL) and 22 mL (15–30 mL); 99.5% of the men had normal pubic hair development (Tanner stage 5 or 6).

### Registry study of testicular cancer

**Figure 3:**
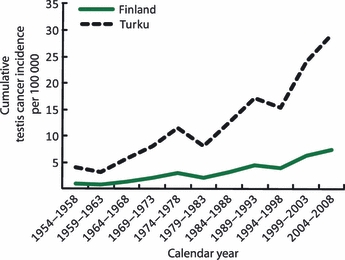
Testis cancer incidences for Finnish men, 10–59 years old at time of diagnosis, according to year of diagnosis and region. Overall, there has been an increasing incidence during the recent 50 years, however, more pronounced in the Turku area where the semen quality studies, illustrated in Figs 1 & 2, have been undertaken.

**Figure 4:**
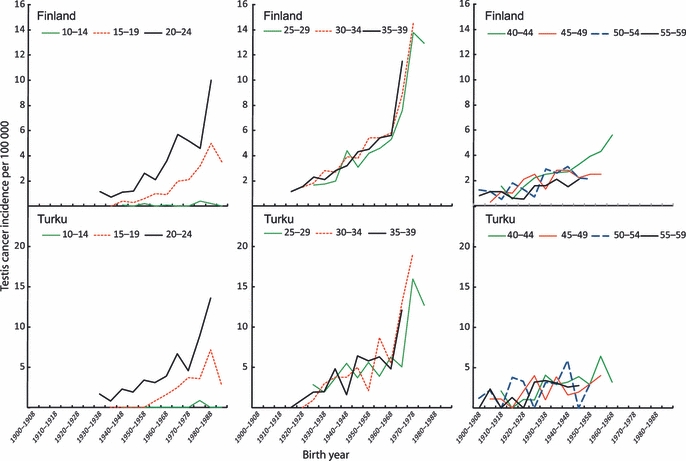
The testis cancer incidences, according to both years of birth and age at time of diagnosis for the cancer patients stratified according to the same geographical regions as in Fig. 3. Except for men aged 10–14 years, the recent birth cohorts have an increased incidence of testicular cancer compared with previous cohorts irrespective of age of cancer diagnosis. However, the results for the most recent cohorts should be interpreted cautiously because of relatively small numbers of men in each sub-group.

Figure 3 shows that the annual testicular cancer incidences from 1954 to 2008 increased more in the Turku area (which is a part of the South-Western area) of Finland than in the entire country. To study the age-dependent temporal trends, we analysed age-adjusted (5-years-age intervals 10–14 years, 15–19 years etc) incidence rates for the entire Finland and the Turku region (Fig. 4). The recent birth cohorts have an increased incidence of testicular cancer compared with previous cohorts irrespective of age of cancer diagnosis, except for men aged 10–14 years.

## Discussion

The investigation of men from the general population of young Finns from the Turku area showed lower sperm counts in the most recent birth cohort compared with only few years older cohort. Additionally, the younger and more recently born men also had higher incidences of testis cancer than the older generations. This increase seemed more pronounced for men from the Turku area where the semen quality study was undertaken than for the entire Finnish population. Thus, the currently young Finnish men in general may suffer from more reproductive health problems than previous cohorts.

We described the detected adverse trends both as a birth cohort phenomenon and as an effect related to the study period. The birth cohort and investigation period are linked, and statistical evaluations cannot help distinguish which is most relevant. However, the risk of testis cancer is linked to birth cohorts (Bergström *et al.*, 1996), and the incidence rate is 8–10 times higher for Finns born around 1980 compared with men born around 1950 (Bray *et al.*, 2006). Furthermore, testicular germ-cell cancers in adults arise from carcinoma-in situ cells which are of foetal origin (Skakkebæk *et al.*, 1987; Jørgensen *et al.*, 1995a,b; Rajpert-De Meyts, 2006; Sonne *et al.*, 2009). Thus, the increase in the testicular cancer incidences should be interpreted as a birth cohort effect.

The described increase for the testicular cancer incidence for the youngest men relies on only a rather small number of men and should therefore be interpreted cautiously. Nevertheless, we find it striking that there seems to be a tendency for an increased risk in all age groups above 15 years old and a higher increase in the Turku area than in the entire country. However, the results can only be regarded as preliminary, and a longer observation period is needed to corroborate or refute whether the indicated trends will last. Nevertheless, the findings can be used as an important indication of the validity of the study hypothesis (e.g. a falsification if trends showed opposite results). Most testicular cancers arise in men 10–60 years-old (Møller, 2001), which we also detected in the current study. A vast majority of tumours in this age-group are germ-cell tumours (>95%) arising from carcinoma-in situ cells, whereas those arising at earlier or later age have a different origin (Jørgensen *et al.* 1995a, Rajpert-De Meyts, 2006). We could not separate the registry information into germ-cell tumours or other testicular tumour types and therefore we chose to show the incidence only for men 10–59 years, which thus can be expected to represent mainly trends for germ-cell tumours.

The TDS concept proposes that an impaired development of foetal testes may lead to an increased risk of cryptorchidism, hypospadias, testicular cancer and decreased spermatogenesis (Skakkebæk *et al.*, 2001), but does not imply that all affected men develop all the four symptoms (Jørgensen *et al.*, 2010). Animal studies illustrate that in utero exposures to anti-androgenic agents may reduce Sertoli cell numbers that are the major factor determining sperm count in an individual (Scott *et al.*, 2007; Sharpe, 2010). Interestingly, a continued post-natal exposure was needed to suppress a post-natal recovery of Sertoli cells after pre-natal exposure to an anti-androgen (Auharek *et al.*, 2010). This indicates that both pre- and post-natal exposures are needed to affect Sertoli cell number permanently, at least if gross abnormalities are otherwise not present. Thus, the low sperm counts may be the result of both pre- and post-natal events.

An increasing number of the young Finns had a sperm concentration below the new WHO reference level of 15 million/mL (World Health Organization, 2010). From a biological point of view, this cut-off level may be too low to indicate normal fecundity (Skakkebæk, 2010) because the chances of achieving a pregnancy decrease with sperm concentrations below 40 million/mL (Bonde *et al.*, 1998). A suggestion of a higher borderline value was also corroborated by a study of European fertile men showing a decrease in waiting time to pregnancy (TTP) with increasing sperm concentration up to 55 million/mL (Slama *et al.*, 2002). An American study showed that only men with a sperm concentration of more than 48 million/mL could be classified as having a normal fertility chance (Guzick *et al.*, 2001). Taken together, this may indicate that the 15% from the latest birth cohort having less than 15 million/mL may get difficulties in fathering children by natural means and that the remaining 28% (difference up to 43%) that have less than 40 million/mL may experience a longer waiting time to pregnancy than men having higher sperm concentrations. It can be argued that total sperm counts better reflect spermatogenesis than sperm concentrations. In the current study, we did not see any change in semen volume and therefore the same conclusions will be reached irrespective of whether sperm concentrations or total sperm counts were investigated.

The high number of morphologically abnormal spermatozoa of the young men also indicated that reduced fecundity may be a frequent problem. According to Guzick *et al.* (Guzick *et al.*, 2001), who used sperm morphology – assessed according to the same criteria as here – a man should have at least 12% normal spermatozoa to be classified as fertile. This was the case only for 26% of the men. van Waart *et al*. detected a reduced pregnancy rate after intra-uterine insemination when the frequency of normal spermatozoa was below 5% (van Waart *et al.*, 2001). Thus, the approximately 22% of the young Finns in our study who had less than 5% normal forms also showed an impaired quality for this important variable for fecundity.

Special caution is needed in the interpretation of the sperm motility data. Sperm motility assessment is highly subjective, and it was not possible for us to undertake a quality control study of the assessment of this variable. However, the distinction between motile and immotile spermatozoa may be more reliable than distinction between different subcategories of motilities (Jørgensen *et al.*, 1997), which is the reason why we have only presented the frequencies of motile spermatozoa.

Only one technician was involved in the study reducing the potential impact by inter-observer variation. The quality control programme showed this technician to assess sperm concentration at a constant level during the years when compared with other participants in the programme. Thus, within-observer variation did not explain the detected trends in sperm count. However, to make the described results directly comparable to those published previously from similar studies from other countries and the initial Finnish study (Jørgensen *et al.*, 2002; Punab *et al.*, 2002; Paasch *et al.*, 2008), a correction has to be applied. These previously published results used the Copenhagen laboratory as reference and adjusted the sperm concentration to the Copenhagen assessment level. Thus, the currently provided results for sperm concentration, total sperm count and total number of morphologically normal spermatozoa should be corrected for the assessment difference of 11% according to the quality control results given in the Materials and methods section (e.g. corrected sperm concentration = observed sperm concentration ×100/111). The effects of potential confounders were accounted for in the statistical analyses, when relevant. The influence of increasing duration of abstinence up to approximately 96 h for semen volume, sperm concentration and total sperm count, and no effect on motility or morphology is in agreement with our initial findings, and with the results of other semen quality studies of men from the Northern European area (Jørgensen *et al.*, 2002; Punab *et al.*, 2002; Paasch *et al.*, 2008). Other studies have shown an adverse effect of maternal smoking during pregnancy on the son's semen quality (Jensen *et al.*, 2004, 2005; Paasch *et al.*, 2008). In contrast, we did not find such an effect here, probably because the number of smoking mothers was very small.

Despite the participation being low, as it often is in studies requesting delivery of semen, we considered the examined men as representative of the normal population of young men from the Turku area. The men had essentially no prior knowledge of their own fertility potential and therefore attention to possible fertility problem is unlikely to have affected their motivation to participate. As shown in Table 3, the same trends in semen parameters was detected when the analysis was based only on men without any known ‘conditions’ as detailed in Table 1. This indicates that such ‘conditions’ were not the reason for the detected trends. The financial compensation received for participating in the study seems unlikely to have led to the selection of men with reduced semen quality. In fact, if compensation had not been given, it is most likely that men suffering from some kind of disease would be more interested in participating – in the hope of receiving advice – than men without diseases. Furthermore, a Danish study evaluating the reproductive hormones FSH, inhibin-B, LH and testosterone detected that the levels of these hormones did not differ between men who delivered semen samples and a larger group of men who only had a blood sample drawn (Andersen *et al.*, 2000). This indicates that men who participate in a semen quality study using our approach do not differ from non-participants when their testicular functions are assessed according to hormonal levels, indicating that the participation rate does not hamper conclusions from the obtained results.

In addition to previously described differences in the testicular cancer rates and semen quality between Finnish men and Danish men (Jørgensen *et al.*, 2002; Chia *et al.*, 2010), we have shown a significant difference in the prevalence of cryptorchidism and hypospadias in boys born in 1997–2001 in Copenhagen and Turku (Virtanen *et al.*, 2001; Boisen *et al.*, 2004, 2005). Danish boys had a higher incidence of these disorders. Furthermore, Finnish boys with normal testicular descent had larger testes and higher inhibin-B values at 3 months of age than healthy Danish boys (Main *et al.*, 2006). These findings suggested to us that we may see similar differences between the countries when the boys mature to adult age; i.e. better semen quality in Finns than in Danes. As the present data on semen quality show deterioration of semen quality in Finnish men born 10–20 years earlier than the 1997–2001 cohort, we might see deterioration also in the Danish group in the future.

The factors causing the adverse trends in male reproductive health remain elusive. However, only environmental factors can explain the rapidly increasing trends in testicular cancer. Factors related to modern way of living may be involved, including exposure to modern industrial chemicals and pesticides. Recent experimental research has revealed that the foetal gonads are particularly vulnerable to endocrine disruption (Auharek *et al.*, 2010; Fisher *et al.*, 2003; Mahood *et al.*, 2005, 2006). Whatever the cause may be, most likely we should search for complex factors operating 20–30 years ago to explain the current reproductive problems of young men. Some Western European countries experienced more rapid post-war industrial development than Finland, which nevertheless more recently has enjoyed a very high economic growth. Today, Finland and other Western European countries are rather similar with regard to industrialization and economy (Statistical Yearbook of Finland, 2006). Our results seem to indicate that Finland will also adapt to these European countries with regard to male reproductive health.

*In conclusion*, our surveillance study revealed that the semen quality of recent birth cohorts of Finnish men seemed to deteriorate. In addition, an increase in the incidence of testis cancer was apparent and more pronounced for men from the Turku area of Finland where the semen studies were undertaken. We speculate that the simultaneous adverse trends in semen quality and testis cancer could be linked through an increasing frequency of men with testicular dysgenesis syndrome among Finnish men. The rapid rate of changes may suggest that the underlying causes are environmental and, as such, preventable. Our findings necessitate not only further surveillance of male reproductive health but also more research efforts to detect and remove the environmental factors which most likely are behind these findings.
